# Irreducible anteromedial radial head fracture-dislocation: value of pre-operative magnetic resonance imaging

**DOI:** 10.1259/bjrcr.20200111

**Published:** 2020-11-17

**Authors:** Lee Kai Lim, Joey Beh

**Affiliations:** 1 Department of Radiology, Ng Teng Fong General Hospital, National University Health System, Singapore, Singapore

## Abstract

We describe a case of an anteromedial fracture-dislocation of the radial head in an adult patient, which was initially irreducible using closed means, and remained challenging to reduce despite open surgery. Further advanced CT/MRI revealed entrapment of the radial head due to the interposition of the brachialis tendon posteriorly, thereby preventing sustained reduction. While three other cases of irreducible anteromedial radial head dislocation due to the brachialis tendon have been reported in the English surgical literature, none of the imaging findings have been described in the radiological literature. Only one other case published in a surgical journal briefly demonstrated pre-operative MRI imaging. We would like to share the value of pre-operative MRI in this rare presentation, which would be helpful in diagnosing not only cases with interposition of the brachialis tendon, but potentially other types of soft tissue interposition which also limit closed reduction. To the best of the authors’ knowledge, this would be the first report on the imaging findings in a radiological journal. Awareness of this phenomenon would assist radiologists in the diagnosis and management of this rare condition.

## Introduction

Radial head fractures are common injuries of the upper limb.^1^ Occasionally, they are complicated by fracture comminution, ulnar fractures, ligamentous injuries and dislocations. Examples of complicated fracture-dislocations include Monteggia fracture-dislocations, Essex-Lopresti lesions, and the terrible triad of injuries. While anteromedial dislocation of the radial head can be seen in Monteggia lesions, isolated anteromedial dislocation and anteromedial fracture-dislocation of the radial head are rare.^2–4^ Within this group of injuries, some are irreducible using closed means therefore requiring open surgical reduction.

A number of soft tissue interposition have been described in the literature which limit radial head reduction, including the anterior joint capsule, the biceps tendon, the annular ligament and the brachialis tendon.^5^ There have been only two cases of brachialis tendon interposition in the surgical literature, while a third case demonstrates buttonholing of the radial head through the brachialis tendon.^2,5,6^ One of these cases briefly mentions pre-operative MRI imaging which was helpful in the diagnosis prior to surgical intervention.^2,5^ We would like to highlight the value of pre-operative MRI in this rare clinical presentation, which would be of interest to both general radiologists and musculoskeletal radiologists.

## Case report

A 58-year-old male patient with a prior history of a surgically treated Arnold Chiari type I malformation presented to the emergency department with right elbow pain and deformity after a fall. He had tripped while coming down the stairs and had landed on his right elbow in a flexed position. Prior to this injury, he had full range of flexion–extension and pronation–supination movements of his right elbow. There was no prior elbow injury.

On examination, he had normal vital signs. A right elbow joint effusion was present, and he was tender along the medial and lateral epicondyles of the right distal humerus. Pain was exacerbated on supination as well as on elbow flexion. The patient had intact neurovascular status distal to the elbow. No wrist or shoulder joint tenderness was reported.

**Figure 1.**
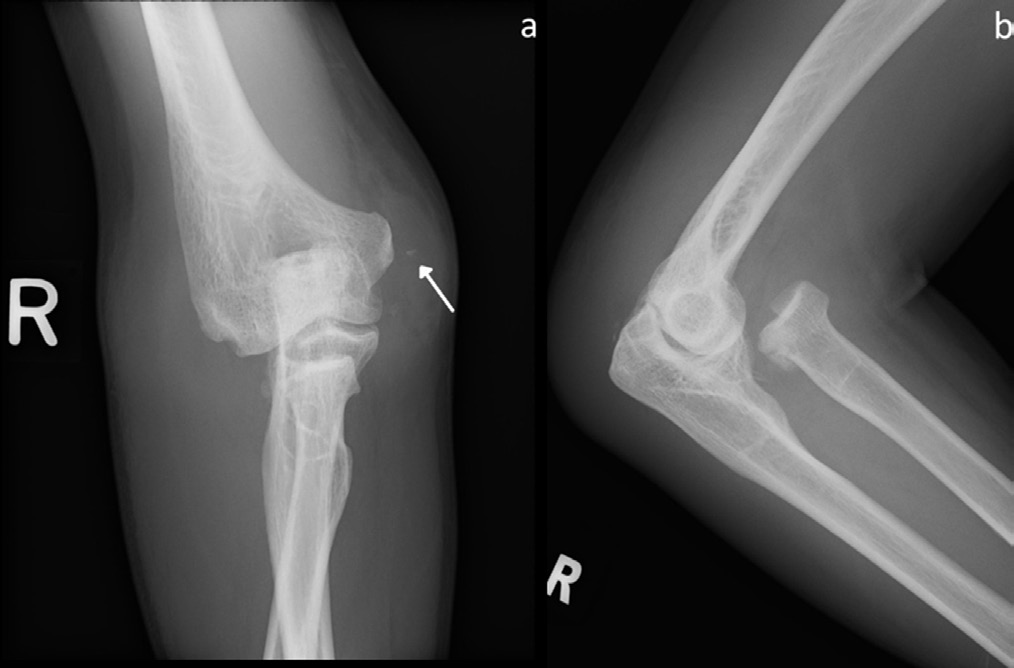
Anteroposterior (a) and lateral (b) plain radiographs of the right elbow in a 58-year-old male patient. There is a comminuted fracture of the radial head, which is also dislocated anteromedially. A tiny avulsed bone fragment is seen adjacent to the medial epicondyle (*arrow*). No ulna fracture is present.

Initial anteroposterior and lateral radiographs of the right elbow demonstrated a mildly displaced fracture of the radial head, which was also dislocated anteromedially relative to the capitellum (Figure 1). No ulna fracture was seen. Attempts to reduce the radial head fracture–dislocation under moderate sedation in the emergency department, followed by closed reduction in the operating theatre under general anaesthesia and fluoroscopic guidance were unsuccessful. Multiple attempts of using posterior directed force to reduce the dislocation were performed but were unsuccessful in maintaining sustained reduction. The attempts were abandoned in view of progressive elbow swelling.

**Figure 2.**
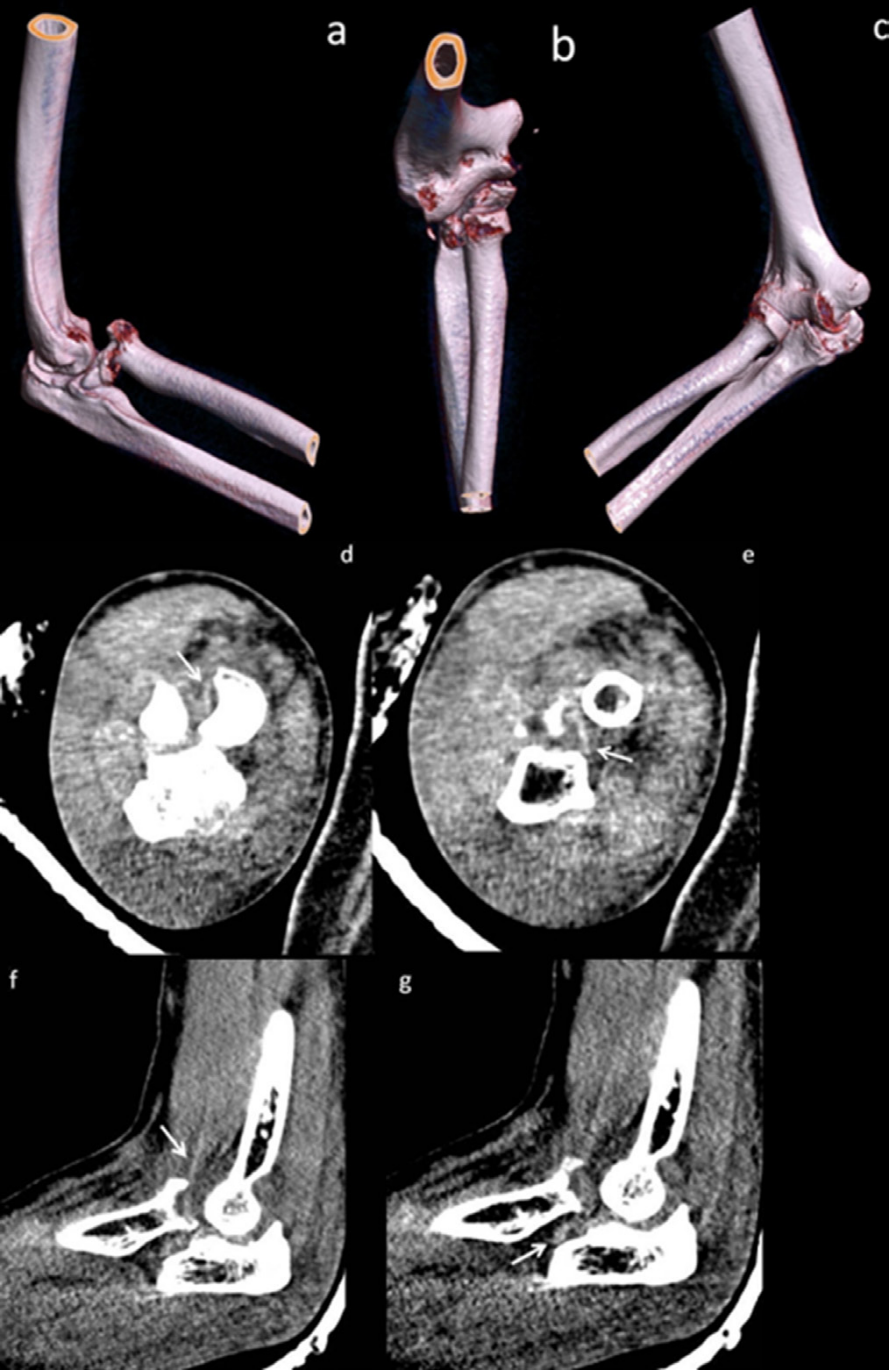
Volume rendered 3D reconstruction images using source data from a CT scan shows the right elbow in the right anterior oblique (a), anteroposterior (b) and left anterior oblique (c) projections. Anteromedial fracture–dislocation of the radial head is again demonstrated. The comminuted radial head fracture comprises about 25% of the radial articular surface. Fracture fragments are seen wedged posterior to the radial head and within the radial notch of the ulna. The ulna trochlear notch is rotated relative to the trochlea of the humerus, suggestive of rotatory subluxation. Axial (d–e) and sagittal (f–g) images in soft tissue windows demonstrate the brachialis tendon (*white arrow*) as a linear hyperdensity traversing posterolateral to the radial head fracture. 3D, three-dimensional.

A CT scan of the right elbow with volume rendered three-dimensional (3D) reconstruction was performed after the examination under anaesthesia (Figure 2). The CT scan confirmed a comminuted fracture of the radial head comprising approximately 25% of the articular surface, which was also dislocated anteromedially relative to the capitellum. Several fracture fragments were wedged posterior to the radial head and within the radial notch of the ulna. The coronoid process was intact, and there was no ulna fracture. The images on soft tissue window were able to demonstrate the brachialis tendon as a faint linear hyperdensity traversing posterolateral to the radial head.

**Figure 3.**
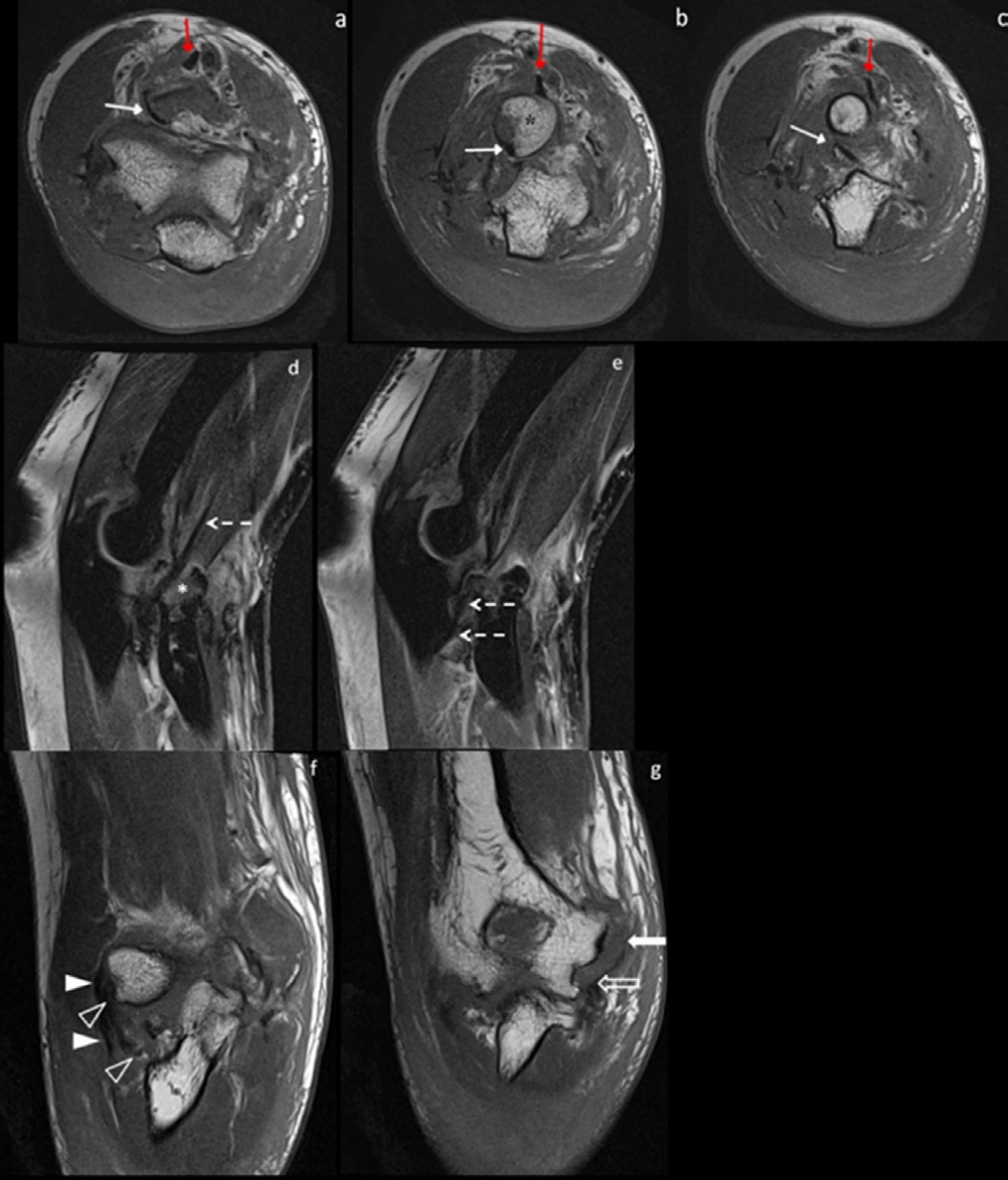
Multiplanar noncontrast MRI scan of the right elbow. (a–c) Axial contiguous *T*_1_ weighted images from proximal to distal across the level of the radial head demonstrate the brachialis tendon (*white arrow*) traversing posterolateral to the radial head (*) to insert into the coronoid process at the tuberosity of the ulna (c). Radial head fracture with associated fragments within the radial notch of the ulna are again seen. The biceps tendon (*red arrow*) is also identified anterior to the radial head. The annular ligament is not seen encircling the radial head, and is completely torn. (d–e) Sagittal Proton Density-weighted fat saturated images of the right elbow demonstrate the brachialis tendon (*Dashed arrows*) along the sagittal plane coursing posterior to the radial head fracture-dislocation (*). (f - g) Coronal *T*_1_ weighted images show disruption of the lateral collateral ligamentous complex in (f) and medial collateral ligaments in (g). (f) Proximal common extensor tendon (*solid arrowhead*) shows high grade partial tear at its origin at the lateral humeral epicondyle and is wavy distally, probably partially torn. The RCL (*open arrowhead*) shows high grade partial tear at its origin at the lateral humeral epicondyle and is completely avulsed from the annular ligament. These findings were confirmed intraoperatively. (g) The medial collateral ligament (*open block arrow*) and common flexor-pronator tendon (*solid block arrow*) show fibers discontinuity at the origin of the medial humeral epicondyle, reminiscent of complete tears. These findings were also confirmed intraoperatively. Extensive subcutaneous, intermuscular and intramuscular oedema are seen across all of the above images. RCL, radial collateral ligament.

The patient subsequently underwent a non-contrast MRI scan of the elbow which demonstrated multiple findings (Figure 3). The most crucial finding involved the brachialis tendon which was located posterolateral to the radial head as it traversed distally to insert into the coronoid process at the tuberosity of the ulna. There was resultant suspension of the radial head upon the brachialis tendon, analogous to a ‘sling’, which hindered anatomical reduction of the radial head posteriorly. Further findings of high grade tears of the lateral ligamentous complex involving the lateral ulnar collateral ligament (LUCL) and radial collateral ligament (RCL) were also seen. The annular ligament was not discretely identified encircling the radial head, and was also completely torn. The medial collateral ligament (MCL) and common flexor-pronator tendon showed discontinuity at their origins at the medial humeral epicondyle, reminiscent of complete tears.

The patient was re-admitted to the operating theatre for surgical reduction, where open reduction was also challenging due to the brachialis tendon interposition. The decision was made by the orthopaedic surgeons to perform a radial head replacement, and repair of the lateral and medial collateral ligaments and annular ligament. Post-operative radiographs of the elbow showed satisfactory implant placement and alignment. The patient was discharged from the hospital with future follow-up in clinic.

## Discussion

Irreducible anteromedial dislocations or anteromedial fracture–dislocations of the radial head are rare.^2,3,5–8^ Ozan et al had summarised the literature on the various types of soft tissue interposition causing irreducible anteromedial radial head dislocation.^5^ The variety of soft tissue interposition which limits closed reduction is fairly broad, ranging from the joint capsule, tendinous structures or the annular ligament.^5^ All cases required open surgical reduction. Ozan et al and Cates et al had reported irreducible anteromedial radial head dislocations secondary to brachialis tendon entrapment, which hindered closed reduction of the radial head thus requiring open surgical reduction.^2,5^ Camp et al also reported involvement of the brachialis tendon in an irreducible anteromedial radiocapitellar dislocation with an associated minimally displaced radial head fracture.^6^ In this case, the radial head had buttonholed through the brachialis tendon which suspended the radial head in a dislocated position.

Pre-operative imaging for this condition had predominantly focused on plain radiographs and CT scans with 3D volume rendered reconstructions. Cates et al briefly illustrated a single pre-operative MRI image which was able to demonstrate the abnormal positioning of the brachialis tendon.^2^ Camp et al and Yoshihara et al suggested that pre-operative MRI imaging may be helpful in the diagnosis of such conditions.^6,9^ The lack of advanced imaging describing this condition in the literature has also been reported by O'Driscoll et al in the surgical text, with suggestions to include MRI, ultrasound or CT imaging as part of the work-up.^10^

This condition and its associated imaging have not been described within the radiological literature. The plain radiographs and CT scans with 3D reconstructions are useful for characterisation of the radial head fractures, exclusion of distal humeral or ulnar fractures, as well as the assessment of radiocapitellar alignment. The CT scan images on soft tissue window were able to faintly demonstrate the brachialis tendon upon close inspection and tracing of the brachialis muscle and its myotendinous junction. This was confirmed on the subsequent MRI.

MRI exquisitely demonstrates the abnormal anatomy and abnormal alignment within the elbow joint. The course of the brachialis tendon is better visualised on axial *T*_1_ weighted imaging as it traverses posterior to the radial head prior to insertion into the ulna. Furthermore, MRI is also able to show the high grade partial and complete tears of the collateral ligamentous complexes and the annular ligament.

Other soft tissue structures apart from the brachialis tendon have been implicated in irreducible radial head anteromedial dislocations. The involvement of the biceps tendon had been reported in the surgical literature.^8,9,11,12^ Various descriptors such as the biceps tendon 'clinging' or 'transposed' around the radial head were used. Veenstra et al had also postulated in 1993 that the radiographic anteromedial position of the radius may be pathognomonic for biceps tendon interposition. However, with the benefit of advanced imaging such as MRI, the biceps tendon is readily identified, as demonstrated in Figure 3. It is highly plausible that MRI would also be able to identify other soft tissue structures, such as the annular ligament, and to formulate a pre-operative diagnosis prior to surgical reduction.

In conclusion, we report a rare case of an irreducible anteromedial fracture–dislocation of the radial head which had benefitted from pre-operative MRI in obtaining a pre-operative diagnosis. Although the CT scan was able to faintly demonstrate the brachialis tendon, MRI provided more exquisite anatomical detail.

Active search for soft tissue interposition (such as the brachialis tendon) on pre-operative MRI together with good clinical history should clinch the diagnosis. MRI finding of brachialis tendon interposition in the context of an irreducible anteromedial fracture–dislocation of the radial head behooves the surgeon to perform open reduction instead of closed reduction which would have been successful in most isolated radial head dislocation cases, as well as prompts the surgeon to actively manage the associated complications such as repair of the annular ligament, the primary stabiliser of the proximal radioulnar joint. In our presented case, the irreducibility of the dislocation despite open surgical techniques highlights the challenging nature of this specific condition. Hence, MRI is helpful in the work-up and should be recommended in such atypical presentations. Awareness of this rare condition in the radiological literature would be helpful for both general and musculoskeletal radiologists in their daily practice.

## Learning points

1. Irreducible anteromedial radial head fracture–dislocation is rare.
2. Soft tissue interposition have been described in the literature which limit radial head reduction, including the anterior joint capsule, the biceps tendon, the annular ligament and the brachialis tendon.
3. Pre-operative imaging have predominantly focused on plain radiographs and CT scans with 3D volume rendered reconstructions.
4. Active search for soft tissue interposition on pre-operative MRI together with good clinical history should clinch the diagnosis.
5. MRI is helpful in the work-up and should be recommended in such atypical presentations.
